# Efficacy of nurse-led *b*reastfeeding *su*pportive *ca*re (B-SUCA) on breastfeeding outcomes among mother-newborn dyads in a tertiary referral hospital of India: a randomized controlled trial

**DOI:** 10.1186/s13006-025-00772-y

**Published:** 2025-11-19

**Authors:** Arkierupaia Shadap, Sonia R.B D’Souza, Shreemathi S. Mayya, Rekha Upadhya

**Affiliations:** 1 Manipal College of Nursing, Manipal Academy of Higher Education (MAHE), Karnataka, 576104 India; 2 SMCON- Sikkim Manipal University, Gangtok, Sikkim 737102 India; 3 Department of Obstetrics and Gynaecological Nursing, Manipal College of Nursing, Manipal Academy of Higher Education (MAHE), Manipal, Karnataka 576104 India; 4 Department of Data Science, Prasanna School of Public Health, Manipal Academy of Higher Education (MAHE), Manipal, Karnataka 576104 India; 5 Department of Obstetrics and Gynaecology, Kasturba Medical College, Manipal Academy of Higher Education (MAHE), Manipal, Karnataka 576104 India

**Keywords:** Breast crawl, SSC, Exclusive breastfeeding, Breastfeeding behaviors, Perinatal outcomes

## Abstract

**Background:**

Despite efforts to promote mother‒newborn skin‒to‒skin contact (SSC) following delivery, this is seldom practiced. World Health Organization (WHO) recommends SSC at least one hour following birth. This trial determined the efficacy of nurse-led *B*reastfeeding *Su*pportive *Ca*re (B-SUCA), an integrated intervention combining breast crawl with SSC for early initiation of breastfeeding outcomes, perinatal outcomes and exclusive breastfeeding (EBF).

**Methods:**

A randomized controlled trial was conducted between 2022 and 2024 at a tertiary referral hospital in India. Approximately 160 mother-newborn dyad’s were enrolled with an allocation ratio of 50:50, with 80 individuals randomly allocated each to intervention and control groups. The intervention group received the intervention in addition to routine standard care provided by the nurse-midwives, and control group received only standard care. Breastfeeding outcomes comprised time to initiation of breastfeeding, breastfeeding behaviors, perinatal outcomes assessed postintervention in the hospital and exclusive breastfeeding from birth to six months assessed by telephonic follow-up.

**Results:**

The median time (in minutes) to initiation of breastfeeding was 32.5 (IQR:25.0–41.0) in the intervention and 48.5(IOR: 44.0 – 54.3) in the control group, respectively. The majority of participants reported positive attachment response (p<0.002), emotional bonding (p<0.001) and swallowing behavior (p<0.002) between the groups. All participants had spontaneous deliveries, and the uterus contracted well following birth. The median time for placental separation in the intervention group was 14.0 (IQR: 11.00 – 15.00) and the control group was 11.0 (IQR: 10.00– 12.30) respectively (p value <0.001). The Apgar scores at 0 min were 6 – 8 (both groups) and at 5 mins (7 – 10) in intervention; (7 – 9) in control group respectively. The mean newborns’ body temperature (°F ) was 98.4 °F (SD = 0.475) intervention group and 98.1 °F (SD = 0.552) control group, with a statistically significant difference (p < 0.001). Sixty-seven (84%) and fifty-six (70%) participants exclusively breastfed up to 6 months in the intervention and control group respectively.

**Conclusion:**

Findings provide critical evidence that nurse-led B-SUCA intervention was effective in improving breastfeeding outcomes. Its integration into routine care can empower nurse-midwives and enhance maternal-infant outcomes.

**Trial registration:**

CTRI Reg. No. is CTRI/2022/03/040974, CTRI Reg. dated 10 March 2022.

## Background

Skin-to-skin contact (SSC) is a crucial evidence-based practice that provides maximized chance for exclusive breastfeeding [1]. A Cochrane review and meta-analysis revealed the benefits of skin-to-skin contact (SSC) in mother‒newborn dyads, and these mothers were more likely to breastfeed at 1‒4 months after birth and exclusively up to 6 months [2]. The World Health Organization (WHO) recommends the practice of SSC for at least one hour after birth [3]. Early initiation of breastfeeding can prevent 22% of all deaths among babies younger than one month in developing countries [4]. Compared with newborn infants who initiated breastfeeding at 2–23 h and 24–96 h after birth, newborn infants who initiated breastfeeding within the first hour after birth had lower neonatal mortality [5]. However, few studies have investigated the success of breastfeeding in the immediate neonatal period [6, 7]. In India, the breastfeeding rate within one hour of birth for children under 3 years of age was 41.6 per the NHFS-4 and 41.8 per the NFHS-5. The exclusive breastfeeding rates for those younger than 6 months were 54.9% according to the NHFS-4 and 63.7% according to the NHFS-5 [8, 9].

Strong scientific research has focused on the importance of the SSC in the first hour after birth. This unique time for both mothers and infants, individually and in relation to each other, provides vital advantages for short‐term and long‐term health, regulation and bonding. However, worldwide, mother-infant SSC in clinical practice lags [10]. Mother–infant SSC also increased the rates of exclusive breastfeeding, which could be used by nurse–midwives and maternal/infant health care providers/personnel to develop evidence-based intervention programs [11].

The United Nations International Children’s Emergency Fund (UNICEF) recommends “breast crawl as the preferred method for mothers to begin breastfeeding their neonates.” It refers to the infant crawling towards the mother’s breast naturally; in this process, establishing SSC between mother–newborn dyads without external interference. The neonate locates the nipple and self-attaches for the first feeding until the first breastfeeding. The Breastfeeding Promotion Network of India (BPNI), Maharashtra, adopted the ‘Breast Crawl’ and recommended this method for the initiation of breastfeeding [12]. The American Academy of Pediatrics (AAP) also recommends that all healthy infants begin skin contact with their mothers immediately after delivery until the first feeding occurs [13].

In middle-income countries of the sub-Saharan African region, the prevalence of SCC was relatively low, but timely initiation of breastfeeding (TIBF) was reported to be high. Significant intercountry variations in the prevalence of SSC and TIBF were also observed, which showed a strong positive association [14]. In a cross-sectional study conducted in Bangladesh, the prevalence of SSC and early initiation of breastfeeding (EIBF) was 16.4% and 70.4%, respectively, which shows that the prevalence of SSC is quite low, whereas the prevalence of EIBF is considered ‘good’ [15]. An infant Breast-Feeding Assessment Tool (IBAT) score before discharge revealed no significant difference in the number of breastfeeding problems encountered during follow-up (30.9 ± 5.51 vs. 32.7 ± 5.84; *P* < 0.25) or in breastfeeding exclusivity (1.50 ± 1.1 vs. 2.10 ± 2.2; *P* < 0.45) [16].

Mothers should be observed for mother‒newborn positioning and attachment at the onset of breastfeeding, and if needed, subsequent counseling should be given on correct positioning and attachment [17]. Notably, several problems related to breastfeeding also prevent successful breastfeeding. A study conducted in India revealed the occurrence of breast fissures, which can deter breastfeeding as the most common problem encountered during breastfeeding, followed by inverted nipples and mastitis, with rates ranging between 65% and 75%, respectively [18]. The combination of wider and longer nipples was also associated with a greater risk for difficulties with proper latching; the combination of wider nipples and denser areolas was also associated with a greater risk for sore nipples [19].

SSC also affects perinatal outcomes, especially in mothers, such as reducing postpartum bleeding, decreasing the time needed to expel the placenta, increasing oxytocin levels that decrease cortisol levels, resulting in happier childbirth experiences, improving bonding with newborns, and increasing confidence in mothers to care for their newborns [20–22]. Despite all the positive outcomes of SSC, a large gap still exists in Indian settings, with more than 50% of the population still not exclusively breastfeeding their infants for the first 6 months as per the duration recommended by the WHO [23].

Therefore, proper planned interventions need to be promoted to increase EBF in Indian mother-newborn dyads. This study aimed to determine the effects of breast crawl and SSC among mother‒newborn dyads in a tertiary referral hospital, which are more supported by one-to-one support from nurse‒midwives immediately during the golden hour of birth. The expected outcomes of the study were the time to initiate breastfeeding, improvement in breastfeeding behaviors, perinatal outcomes and long-term effects such as exclusive breastfeeding rates among mother‒newborn dyads for the first six months following birth. The present study also aimed to contribute to the Sustainable Development Goals (SDGs) to end poverty in all its forms everywhere; zero hunger; ensure healthy lives; promote well-being for all at all ages; and revitalize the global partnership for sustainable development.

## Methods

### Study design and period

**Fig. 1:**
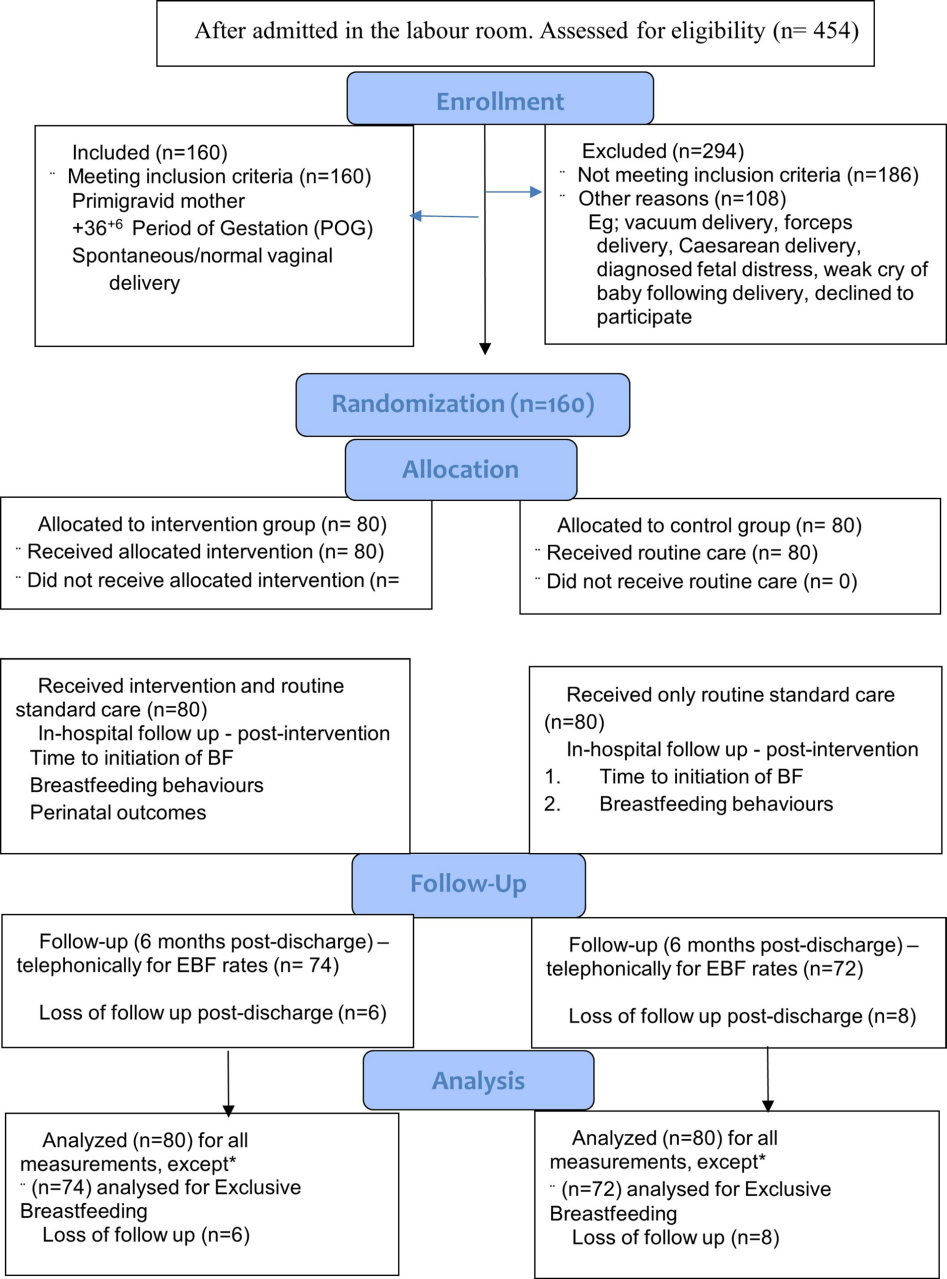
CONSORT 2025 flow diagram of the participants

This trial is an open-label randomized controlled trial (RCT) with an intervention and a control group, with participants being individually allocated to either of the groups [24]. The allocation ratio was 50:50 and is depicted by a CONSORT 2025 flow of the participants (Fig. 1) [25]. The study was conducted between November 2022 and October 2024 among 160 mother-newborn dyads (80 participants each allotted to the intervention and control groups) admitted to the labor wards of a tertiary referral hospital in Sikkim, India. The participants included were primigravid mothers at + 36^+6^ Period of Gestation (PoG) with spontaneous normal vaginal delivery. The sample size calculation was performed using the following formula:

$$\:\text{n}=\frac{2{\left({\text{Z}}_{1-\raisebox{1ex}{${\upalpha\:}$}\!\left/\:\!\raisebox{-1ex}{$2$}\right.}+{\text{Z}}_{1-{\upbeta\:}}\right)}^{2}}{{{\Delta\:}}^{2}}$$

$$\mathrm n\;=\;\mathrm{sample}\;\mathrm{size}\;\mathrm{in}\;\mathrm{each}\;\mathrm{group}$$

$$Z_{1-\;\alpha/2\;}=1.96\;\mathrm{at}\;\mathrm{the}\;5\%\;\mathrm{level}\;\mathrm{of}\;\mathrm{significance}$$

$${\mathrm Z}_{1\;-\;\mathrm\beta}\;=\;0.84\;\mathrm{at}\;80\%\;\mathrm{power}$$

$$\triangle\;=\;\mathrm{effect}\;\mathrm{size}\;=\;0.5\;(\mathrm{medium}\;\mathrm{effect})$$

The researchers adopted *block randomization* with blocks of varying sizes (8, 10,12). A staff member who was not involved in the study generated the random sequence with the help of Research Randomizer, an online random number generator. Both the researcher and the participants were blinded to the random assignment of the participants to the intervention and control groups. A staff member who was not involved in the study allocated the participants using sequentially numbered, opaque, sealed envelopes (SNOSEs) for allocation concealment. This study was conducted as an open-label RCT due to the nature of the intervention— nurse-led breastfeeding supportive care (B-SUCA)—which inherently involves direct interaction between the intervention providers (nurse-midwives) and the participants (mothers). Therefore, the blinding of participants and care providers was not feasible.

The B-SUCA intervention included personalized, real-time support and education provided by the researcher as well as trained nurse-midwives, which was visible and distinguishable from routine standard care. This made it impractical to conceal group allocation from those assessing outcomes, especially when assessments involved direct observation or interviews.

The researcher and the nurse-midwives were also involved in delivering the intervention and were also responsible for documenting outcomes such as the time of initiation of breastfeeding and early feeding behaviors, as these were part of routine clinical records and care processes. Moreover, recruiting separate blinded assessors for each mother‒newborn dyad in a busy tertiary care setting posed significant logistical and resource challenges, which could have compromised the feasibility and sustainability of the study. To minimize potential bias and avoid harm, the study prioritized the use of objective and verifiable outcome measures assessed via valid and reliable tools.

### Procedures

The participants in both the intervention and control groups received routine standard care as per hospital policies. The participants in the intervention group received nurse-led breastfeeding supportive care (B-SUCA) intervention in addition to routine standard care. The B-SUCA intervention included breast crawl and SSC, which were implemented by the nurse-midwives immediately after the delivery of the baby. The participants in both groups were informed regarding the intervention via a participant information sheet (PIS), and free informed written consent was obtained. After random allocation, the participants allocated to the intervention group were again informed explicitly regarding the intervention before it was administered by the nurse-midwives. The outcome measurements were assessed (in-hospital) during the time the mother‒newborn dyads were admitted to the labor delivery recovery rooms (LDRs) and postnatal wards of the hospital. However, the follow-up for both groups regarding exclusive breastfeeding was performed at 6 months post-discharge through telephone follow-up.

### Study measures

The tools used to measure outcomes were subjected to content validation performed by subject experts, pretested and checked for reliability in the setting. The tools used were the baseline demographic and obstetrical characteristics, the observation checklist for the time to initiation of breastfeeding, the Breastfeeding Behavior Assessment (BFBA) tool, the exclusively breastfeeding follow-up tool and the perinatal outcomes tool.

#### Baseline demographic and obstetrical characteristics

Were obtained by interviewing the mothers and abstracting the medical records data of the participants.

#### The time of initiation of breastfeeding (observational checklist)

Was assessed after the delivery of the baby by the nurse-midwives.

#### Breastfeeding behaviors

Were assessed via the Breastfeeding Behavior Assessment (BFHA) in both groups after delivery and postintervention by nurse-midwives who were trained to assess breastfeeding behavior by the researcher. BFBA is a valid and reliable tool that has five components or domains. They are the positioning of the baby to the breast, attachment responses, emotional bonding, health of the breast(s) and swallowing behavior during breastfeeding. Each component has multiple positive and negative items. Each item has a response of ‘Yes’ or ‘No’. Each positive item is scored for ‘Yes’ as ‘1’ and ‘No’ as ‘0’, whereas each negative item is reverse scored: Yes’ is ‘0’ and ‘No’ is ‘1’. The total scores for each of the components or domains of breastfeeding behaviors assessed via the BFBA are as follows: Positioning of baby to breast has 10 items with positive scores from 1–5 and negative scores from 6–10; Attachment responses have 12 items with positive scores from 1–6 and negative scores from 7–12; emotional bonding has 7 items with positive scores from 1–3 and negative scores from 4–7; health of the breast has 7 items with positive scores from 1–3 and negative scores from 4–7; and swallowing behavior during breastfeeding has 16 items with positive scores from 1–8 and negative scores from 9–16.

#### Perinatal outcomes

Were assessed during and postdelivery through an observation checklist and the extraction of medical records data of mother-newborn dyads from both groups.

#### Exclusive breastfeeding

Exclusive breastfeeding up to six months of age was confirmed telephonically for each participant post-discharge in both groups, with the participants confirming that the infant was feeding wholly on breastmilk for the past 6 months. A dichotomous response of ‘Yes’ or ‘No’ was used to record their responses.

The outcome assessments were performed by the researcher and nurse-midwives postintervention and during follow-up post discharge without any blinding; however, allocation concealment was performed during random allocation of the participants to the intervention and control groups via SNOSEs.

### Ethical considerations

Ethical clearance for the study was obtained from the Institutional Research Committee (IRC) and Institutional Ethics Committee (IEC) of the tertiary referral hospital, and the trial was registered in the Clinical Trials Registry of India (CTRI Reg. No. is CTRI/2022/03/040974, CTRI Reg. dated on 10 March 2022). A participant information sheet (PIS) and free informed written consent were obtained from all the participants.

### Statistical analysis

The baseline demographic characteristics were summarized via descriptive statistics, and statistical significance was set at *p* < 0.05 for both the intervention and control groups. The frequency percentage, mean, standard deviation (SD), and chi-square test were applied for categorical data, and the Mann‒Whitney U test was used for continuous data to compare the differences among the groups. There were no missing data; however, the data of few participants in both groups during the 6-month follow-up for EBF rate assessment were excluded from the analysis. The data were computed and analyzed via *Jamovi*. (Version 2.6) [26].

## Results

Study recruitment started in November 2022 and was completed in October 2024. The researcher screened 454 primigravidae for eligibility and recruited 160. These 160 participants were then allotted through a block randomization technique—80 each into the intervention and control groups. The baseline characteristics of participants in the intervention (*n* = 80) and control groups (*n* = 80) were similar (see Table 1).

**Table 1:** Baseline demographic characteristics of participants in the intervention and control groups

			**(N=160)**
**Section A:** **Baseline demographic characteristics**	**Intervention Group (n = 80)**	**Control Group (n=80)**	**p value**
	**M±SD**	**M±SD**	-
	26.8±5.09	26.9±4.98	
Age (in years)	**f (%)**	** f (%)**	0.515
18–27	**46 (57.5)**	**40(50)**	
28 and above	34 (42.75)	40(50)	
Religion			
Christian	15(18.8)	10(12.5)	0.412
Hindu	**44(55)**	**45(56.3)**	
Buddhist	18(22.5)	24(30.0)	
Muslim	3(3.75)	1(1.25)	
Educational qualification			0.883
No formal education	12(15)	13(16.25)	
Primary-high school	**34(42.5)**	**29(36.3)**	
Higher secondary school	25(31.25)	28(35)	
Graduate-Postgraduate	9(11.25)	10(12.5)	
Income (Rs.)			0.672
<10,000	12(15)	12(15)	
≥10,000–30,000	**32(40)**	25(31.25)	
≥30,001–50,000	25(31.25)	**31(38.8)**	
≥50,001	11(13.75)	12(15)	
Occupation			0.196
Housewife	19(23.75)	22(27.5)	
Health care Professional	20(25)	28(35)	
Non-Health care Professional	**41(51.3)**	**30(37.5)**	
Residence			0.634
Urban	35(43.75)	38(47.5)	
Rural	**45(56.3)**	** 42(52.5)**	
Section B:Source of information			
Heard about breastfeeding	**80 (100)**	**80 (100)**	-
Health care professional	70(87.5)	**80 (100)**	0.376
Family	41(51.3)	39 (48.8)	0.209
Friends	68 (85)	73 (91.2)	0.141
Media	8 (10)	70(87.5)	0.377
Mother’s Height (cm)	**M±SD**	**M±SD**	-
	156.85**±**3.67	158.74**±**4	-
Mother’s Weight (kgs)	62.96**±**5.0	61.04**±**4.9	-
Gestational age (weeks)	37±1.2	38±0.5	-
Minimum number of antenatal visits	1.86**±**0.4	1.89**±**0.3	-

*M* Mean, *SD* Standard deviation

The median time (in minutes) to initiation of breastfeeding was 32.5 (IQR:25.0–41.0) in the intervention and 48.5(IOR: 44.0–54.3) in the control group, respectively. Since the data did not follow normality, a Mann‒Whitney U test was performed, which revealed a statistically significant difference on time to initiation of breastfeeding between the intervention and control groups, suggesting that the B-SUCA intervention was associated with a shorter time to initiation of breastfeeding (see Table 2).

**Table 2:** Time to initiation of breastfeeding among mother-newborn dyads in the intervention and control groups

						(N = 160)
	Group	*n*	Median	Interquartile range (IQR)	Mann‒Whitney U test - Statistic	*p* value
Time to initiation of breastfeeding	Intervention	80	32.5	25.0–41.0	1031	<0.001*
	Control	80	48.5	44.0–54.3		

*p < 0.001

The researchers also assessed breastfeeding behaviors postintervention until the mother was in the hospital. The breastfeeding behaviors of the control group, who received only routine standard care, were also assessed. A mean rank comparison of each component of the breastfeeding behaviors was performed to compare the groups. The mean rank comparison of the five domains/components of positive breastfeeding behaviors was greater in the intervention group than in the control group (see Table 3).

**Table 3:** Estimates of the mean rankings, standard deviations and Mann‒Whitney U tests of breastfeeding behaviors among mother-newborn dyads in the intervention and control groups

						[N = 160]
Ranking of each component of breastfeeding behavior	Group	*N*	Mean Rank	Standard Deviation	Mann -Whitney U Statistic	*P* Value
Positioning of baby to breast during breastfeeding	Intervention	80	88.24	45.03	2581	0.023
	Control	80	72.76	39.60		
Attachment responses during breastfeeding	Intervention	80	90.46	42.53	2404	0.002*
	Control	80	70.54	35.15		
Emotional bonding during breastfeeding	Intervention	80	93.43	35.75	2166	< 0.001*
	Control	80	67.58	41.51		
Health of breast during breastfeeding	Intervention	80	83.75	25.13	2940	0.146
	Control	80	77.25	30.86		
Swallowing behavior during breastfeeding	Intervention	80	90.71	35.83	2384	0.002*
	Control	80	70.29	43.14		

*p < 0.001

The frequency and percentage distribution of each item under the major domains/components of breastfeeding behaviors are crucial, as they compare the rate and how much differences in the breastfeeding behaviors pertaining to each item appear between the participants in the intervention and control groups. Compared with those in the control group, the majority of the intervention group who received the B-SUCA intervention displayed better positive breastfeeding behaviors, as shown by their receiving positive scores pertaining to each of the domains/components of the breastfeeding behaviors. However, a minimal number of mothers displayed negative breastfeeding behavior during breastfeeding. The participants were first-time mothers, which might be the reason for their negative breastfeeding behaviors (see Table 4).

**Table 4:** Estimate of domain/component-wise breastfeeding behaviors among the groups

		[*N* = 160]
Components of Breastfeeding behavior assessment	Intervention Group, *n* = 80	Control Group, *n* = 80
	Yes f (%)	Yes f (%)
I. Positioning of baby to breast during breastfeeding		
1. Mother relaxed and comfortable	73(91)	62(78)
2. Baby’s body close, facing breast	77(96)	67(84)
3. Baby’s head and body straight	80(100)	78(98)
4. Baby’s chin touching breast	79(99)	78(98)
5. Baby’s bottom supported	72(90)	76(95)
6. Shoulders tense, leans over the baby	15(19)	5(6)
7. Baby’s body away from mother’s	7(9)	9(11)
8. Baby’s neck twisted	15(19)	10(13)
9. Baby’s chin not reaching breast	35(44)	15(19)
10. Only shoulder or head supported	8(10)	4(5)
II. Attachment responses during breastfeeding	**Yes ** **f (%)**	**Yes ** **f (%)**
1. Baby reaches for breast if hungry	69(86)	74(93)
2. Baby roots for breast	78(98)	72(90)
3. Baby explores breast with tongue	80(100)	77(96)
4. Baby calm and alert at breast	79(99)	79(99)
5. Baby stays attached to breast	78(98)	76(95)
6. Signs of milk ejection, (leaking, after-pains)	72(90)	69(86)
7. No response to breast	5(6)	7(9)
8. No rooting observed	15(19)	13(16)
9. Baby not interested in breast	31(39)	6(8)
10. Baby restless or crying	12(15)	15(19)
11. Baby slips off breast	8(10)	10(13)
12. No sighs of milk ejection.	14(18)	13(16)
III. Emotional bonding during breastfeeding	**Yes ** **f (%)**	**Yes ** **f (%)**
1. Secure, confident hold	72(90)	55(69)
2. Face to face attention from mother	79(99)	77(96)
3. Much touching by mother	77(96)	76(95)
4. Nervous or limp hold	7(9)	8(10)
5. No mother/baby eye contact	14(18)	9(11)
6. Little touching	5(6)	8(10)
7. Shaking or poking baby	4(5)	5(6)
IV. Health of breast during breastfeeding	**Yes ** **f (%)**	**Yes ** **f (%)**
1. Breast softens after feed	76(95)	70(88)
2. Nipples stand out, protractile	77(96)	77(96)
3. Skin appears healthy breast looks round	77(96)	74(93)
4. Breast engorged	4(5)	10(13)
5. Nipple flat or inverted	2(3)	5(6)
6. Fissures or redness of skin	4(5)	3(4)
7. Breast looks stretched or pulled	4(5)	4(5)
V. Swallowing behavior during breastfeeding	**Yes ** **f (%)**	**Yes ** **f (%)**
1. Mouth wide open	76(95)	61(88)
2. Lower lip turned outward	78(98)	77(96)
3. Tongue cupped around breast	76(95)	78(93)
4. Cheeks round	76(95)	75(94)
5. More areola above baby’s mouth	78(98)	73(91)
6. Slow deep sucks, burst with pauses	75(94)	74(93)
7. Can see or hear swallowing	68(85)	72(90)
8. Baby releases breast	66(83)	62(76)
9. Mouth not wide open, points forward	8(10)	10(96)
10. Lower lip turned in	5(6.3)	4(5)
11. Baby’s tongue not seen	6(8)	5(6.3)
12. Cheeks tense or pulled in	3(4)	5(6.3)
13. More areola below baby’s mouth	13(16)	10(12.5)
14. Rapid sucks only	5(6.3)	7(9)
15. Can hear smacking or clicking	14(18)	9(11.3)
16. Mother take baby off breast	14(18)	12(15)

The study also measures the duration of newborn suckling the breast per feeding (in minutes), which is the duration of breastfeeding among mother‒newborn dyads in the intervention and control groups. The duration of breastfeeding (in minutes) ranged between 8 and 22 in the intervention group and 5–25 in the control group. However, there was no statistically significant difference in the duration of breastfeeding between the two groups (see Table 5).

**Table 5:** Duration of newborn suckling the breast per feeding (in minutes) among the mother‒newborn dyads in the intervention and control groups

						[*N* = 160]
Group	n	Range of scores		Median (Q1, Q3)	Mann‒Whitney U test	p value
		Min.	Max.			
Intervention	80	8	22	14.0 (10.0, 17.0)	3054	0.614
Control	80	5	25	15.0 (10.0, 18.0)		

The researcher encountered loss to follow-up among 6 (7.5%) and 8 (10%) in the intervention and control groups, respectively. The actual rates of EBF are 67 (84%) in intervention and 56 (70%) in the control group. However, there were no statistically significant differences between the intervention and control groups with respect to EBF. The data were computed and analyzed as per objectives (see Table 6) [24].

**Table 6:** Estimated exclusive breastfeeding (EBF) rates among the participants in the intervention and control groups.

					[N = 146]
Exclusive breastfeeding rates	Intervention Group *n* = 74 Yes - f (%)	Control Group *n* = 72 Yes - f (%)	χ 2	df	*p* value
At 6 months (24 weeks)	67 (84%)	56 (70%)	4.79	2	0.091

χ2 = chi-square, df = degree of freedom

The perinatal outcomes of the mother‒newborn dyads postintervention in both the intervention and control groups were depicted descriptively. The summary measures show that all participants had spontaneous deliveries, and the uterus contracted well following birth, which was a normal finding observed in both groups. The median time in minutes for placental separation in the intervention group was 14.0 (IQR: 11.00–15.00) and the control group was 11.0 (IQR: 10.00–12.30) respectively (p value < 0.001). (see Table 7).

**Table 7:** Summary measures of perinatal outcomes in the intervention and control groups. [*N* = 160]

						[N = 160]
Perinatal Outcomes		Intervention Group *n* = 80		Control Group *n* = 80	χ2_(df=1)_	*p*-value
(Maternal Outcome before/during delivery)		f (%)		f (%)		
Rise in blood pressure > 130/90 mmHg		23(28.8)		19(24)	0.517	0.472
Induction of labor – spontaneous		57(71.3)		51(63.8)	6.14	0.013
Preeclampsia		6(8)		3(4)	1.06	0.303
Perineal tear		7(9)		3(4)	1.71	0.191
Blood transfusion		2(3)		3(4)	0.206	0.650
Delivery spontaneous		80(100)		80(100)	-	-
Uterine consistency after delivery- Hard		80 (100)		80(100)	-	-
Total blood loss < 500(in ml approx.)		78(97.5)		75 (94)	1.34	0.246
Post-Partum Hemorrhage (PPH)		2(2.5)		0	2.03	0.155
Maternal Outcome postdelivery		**n**	**Median**	**Interquartile range (IOR)**		**p value**
Time taken for placental separation (in mins)	Intervention	80	14.0	11.00–15.00		0.001*
	Control	80	11.0	10.00–12.30		

The summary measures depicted in Table 8 show the perinatal outcomes pertaining to the newborns in both the intervention and control groups, which show significant differences in frequency, percentage, chi-square and p-values. All newborns were born at term (> 37 weeks of gestation), cried immediately after delivery, and were appropriate for gestational age (AGA). Five newborns in each group required brief neonatal intensive care unit (NICU) admission, postintervention in the intervention group for observation. Upon discharge from the NICU, they were reunited with their mothers.

**Table 8:** Summary measures of perinatal outcomes in newborns born to participants in the intervention and control groups

						[N = 160]
Perinatal Outcomes Newborn Outcomes during and post delivery		Intervention Group *n* = 80 f (%)	Control Group *n* = 80 f (%)		χ2_(df=1)_	*p* value
Cried immediately after birth		80(100)	80(100)		-	-
Meconium pass < 24 h		64(80)	60(75)		0.573	0.449
Sign of Jaundice		13(16)	14(17.5)		0.044	0.833
NICU admission required		5(6.3)	5(6.3)		0.00	1.000
Neonatal death		0	0		-	-
Newborn Outcomes during and post delivery	**Group**	**n**	**Range score** **Min. - Max.**		**Median (Percentile) score**	**Mann-Whitny U test** ***p Value***
					**Median (Q1**,** Q3)**	
APGAR score at 0 min	Intervention	80	6	8	7.00 (7.0,7.00)	0.112
	Control	80	6	8	7.00 (7.0,7.00)	
APGAR score at 5 min	Intervention	80	7	10	8.00 (8.00,9.00)	0.289
	Control	80	7	9	8.00 (8.00,8.00)	
Newborn’s body temperature (degrees F)		**n**	**Mean**		**Standard deviation**	**Mann-Whitny U test** ***p Value***
	Intervention	80	98.4		0.475	0.001*
	Control	80	98.1		0.552	

*p < 0.001

Chi-square tests revealed no significant differences in neonatal outcomes between the intervention and control groups. Along with median and percentile values, Mann‒Whitney tests were used to analyze APGAR scores at 0 min and 5 min. The mean newborns’ body temperature (°F) was higher i.e., 98.4 °F (SD = 0.475) in the intervention group and 98.1 °F (SD = 0.552) in the control group, with a statistically significant difference (*p* < 0.001). (see Table 8).

## Discussion

### Time to initiation of breastfeeding

B-SUCA intervention significantly improved the time to initiation of breastfeeding. A cross-sectional study conducted in sub-Saharan African region support these findings, where mothers practicing SSC were more likely to initiate early breastfeeding [aOR = 1.68, 95% CI = 1.58, 1.78]. The pooled prevalence of SSC and timely initiation of breastfeeding were 45.68% (95% CI = 34.12–57.23) and 62.89% (95% CI = 55.67–70.11), respectively [14]. Another study, also in line with the findings of the present study, reports that newborns who received SSC initiated breastfeeding within 2.41 ± 1.38 (M ± SD) minutes after birth; compared to those who received routine care 5.48 ± 5.7 (M ± SD) minutes [1]. One study reported successful breast crawl and first feeding within 60 min, though some minimal assistance for positioning and latching was required [27]. Another study on the effectiveness of SSC at birth on neonatal outcomes among parturients revealed that SSC between mothers and babies at birth improved the initiation of breastfeeding and improved the quality of first breastfeeding among parturient(s) in the intervention group (>0.05 level) [28]. While most studies affirm the positive impact of SSC, one prospective cohort study done among 163 neonates found no statistically significant differences in the time of initiation of breastfeeding (*P =* 0.154) [29].

### Breastfeeding behaviors

Participants in the intervention group reported positive breastfeeding behaviors when compared to those in the control group, who experienced more difficulties like engorgement was reported by 13% compared to 5% in the intervention group; flat or inverted nipples by 6% in control and 3% in the intervention group. Participants in both groups had equal reports of stretched or pulled breasts (5% in each group); fissures or redness of skin were noted in 4% in control and 5% in the intervention group.

One study that assessed breastfeeding practices noted that 38.1% of the mothers had poor breastfeeding positioning [30]. Another study comparing mothers (*n* = 207) with breast problems - cases and without breast problems - controls (*n* = 359) found approximately 146 (70%) had breast fissures, 89 (43%) mothers had inverted nipples, and 46 (22%) mothers had mastitis [31] in the case group. However, one RCT refutes the findings of the present study, suggesting that no significant differences were noted in the intervention and control groups with respect to the Breast Assessment Tool (BAT) scores (median: 8, interquartile range (IQR) 5–10 vs. median 9, IQR 5–10; *p* = 0.6) [32].

### Total duration of breastfeeding per feed (in minutes)

One study reported that ineffective techniques were significantly associated with mothers reporting early breastfeeding problems, which may influence breastfeeding duration [33]. Another study reported a statistically significant difference in the duration of first breastfeeding (*p* < 0.001) [34], which refute the findings of the present study.

### Exclusive breastfeeding (EBF) rates

EBF rates varied across studies. One study reported EBF rates at 7 days, 42 days and 5 months post-partum being 61.7%, 64.6%, and 55.8%, respectively, with no significant differences in the feeding patterns between the successful and failed breast crawl groups during the follow-up period (*P >* 0.05) [34]. In contrast, two RCTs revealed significantly higher EBF rates in early-SSC group at 48 h and at 6 weeks postpartum compared to the control group (95.0 vs. 38.1%; relative risk (RR): 2.5, 95% confidence interval (95% CI): 1.4–4.3 and 90 vs. 28.6%; RR: 3.2, 95% CI: 1.6–6.3) [32]. Rates of EBF were significantly greater at 6 weeks of age in the SSC group compared to the control group (72% vs. 57.6%, *p* < 0.04; relative risk: 1.3; 95% confidence interval: 1.0–1.6) [35]. However, one study reported low EBF rates (8.4%) for infants younger than 6 months, and only 50.9% of the infants were breastfed [36].

### Perinatal outcomes

B-SUCA intervention significantly improved newborn body temperature (mean: 92.66 °F vs. 83.34 °F; *p* < 0.001) in the intervention and control groups, respectively. Supporting studies show lower hypothermia prevalence in SSC groups (2% vs. 42%) and significantly higher axillary temperature, heart rate, and respiratory rate in SSC infants (*p* < 0.0001) [1]. Another study shows the mean axillary temperature, heart rate and respiratory rate of neonates were greater than those in the control group (but within the normal range), and statistically significant differences (*p* < 0.0001, 0.0001 & 0.0001) were noted between the two groups [37]. One study reported similar findings in terms of placental separation time (*p* = 0.0001), which in turn was effective by initiating breastfeeding [38]. Another study on the breast crawl effect supports the present study findings, reporting that 19 (63%) mothers in the intervention group had ≤ 6 min duration of placental separation in the third stage of labor [39]. Related findings also indicate reduced postpartum blood loss in SSC groups [40]. Another study revealed 22% lower risk of composite Post Partum Hemorrhage (PPH) morbidity among mothers who received SSC (adjusted relative risk 0.78, 95% CI 0.65–0.92) [41].

### Strengths

B-SUCA, is an integrated intervention using innovative approach of both breast crawl as well as SSC, implemented in the northeastern region of India, and the findings provide strong evidence supporting the routine implementation of such nurse-midwives’ led interventions specifically in the labor rooms/theatres immediately following childbirth.

### Limitations

Firstly, the present study was conducted at a single center; the outcome measurements and the outcome assessors were not blinded, and the perinatal outcomes were reviewed from the records. Secondly, the present study was limited to only primigravidae, who had spontaneous normal vaginal delivery; however, mothers with cesarean section were excluded, which makes the generalization of results limited to the population of the study. Thirdly, we faced a follow up challenge to gather data on exclusive breastfeeding rates from the participants. They were assessed only through telephonic communication, risking loss to follow up. Finally, comparisons of select perinatal outcomes at six months and twelve months post-intervention could not be included as outcome assessments due to logistical difficulties and time constraints. This may limit the assessment of the interventions’ long-term effects.

## Conclusions

The B-SUCA intervention proved to be successful in decreasing the time to initiation of breastfeeding and improving breastfeeding behaviors, which are crucial for establishing successful breastfeeding, improving EBF rates and contributing to numerous benefits for both the mother(s) and their newborn(s). The effect is also evident with regard to perinatal outcomes in both mothers and their newborns.

Although many studies support the effects of breast crawl and SSC, midwives and other healthcare personnel fail to recognize and implement this method in their daily care for mother‒newborn dyads. Thus, there is still a need to conduct further studies to strengthen the effect of breast crawl and SSC for their long-term effects on mother–newborn dyads, the findings of which may provide valuable opportunities for mothers to improve exclusive breastfeeding.

The present study contributes to the four United Nations Sustainable Development Goals (SDGs), first, in terms of poverty, whereby promoting early breastfeeding through breast crawl and SSC can reduce healthcare costs and improve long-term health outcomes, which can help break the cycle of poverty, especially in low-income communities; second, zero hunger, where breastfeeding is a critical component of food security for infants that provides essential nutrients for the first six months of life; third, good health and well-being, where breastfeeding supports the health and well-being of both mothers and newborns; and fourth, partnerships for the goals, where this intervention, which is performed in collaboration with other healthcare personnel (e.g., consultants, midwives, nurses), is essential for promoting and supporting breastfeeding practices that strengthen healthcare systems and improve outcomes.

## Data Availability

No datasets were generated or analysed during the current study.
